# *In vitro* Antimicrobial Activity of Ampicillin-Ceftriaxone and Ampicillin-Ertapenem Combinations Against Clinical Isolates of *Enterococcus faecalis* with High Levels of Aminoglycoside Resistance

**DOI:** 10.2174/1874285800802010079

**Published:** 2008-06-09

**Authors:** Maria Bruna Pasticci, Antonella Mencacci, Amedeo Moretti, Nicola Palladino, Luigi Maria Lapalorcia, Francesco Bistoni, Franco Baldelli

**Affiliations:** 1 Infectious Disease Section, Department of Experimental Medicine and Biochemical Sciences, University of Perugia, Perugia, Italy; 2 Microbiology Section, Department of Experimental Medicine and Biochemical Sciences, University of Perugia, Perugia, Italy

## Abstract

This paper reports on the in vitro antimicrobial activity of ampicillin-ceftriaxone and ampicillin-ertapenem combinations against five strains of *E. faecalis* with high-level aminoglycoside resistance recovered from blood of septicemic patients. Double disk diffusion test and time killing curves were used. A bacteriostatic synergistic effect between ampicillin and ceftriaxone was detected using the disk diffusion assay for three of the five enterococcal strains studied. With the same three isolates enhanced bactericidal activity was also observed using time killing experiments. Overall, for these three strains, after 24 hr of contact, a decrease ≥ 2 log_10_ from the initial bacterial inoculum was registered with most ampicillin-ceftriaxone combinations, reaching with some of them a colony reduction ≥ 3 log_10_. This bactericidal interaction was negatively influenced increasing the bacterial inoculum. In all five isolates neither a bacteriostatic nor a bactericidal cooperation was observed for ampicillin combined with 2 mg/l of ertapenem.

This investigation broadened the evidence of antimicrobial synergism in vitro between ampicillin and ceftriaxone in selected strains of *Enterococcus faecalis *with high-level aminoglycoside resistance.

## BACKGROUND

Enterococci have been reported as major cause of acquired nosocomial infections including urinary tract infections, intra-abdominal and pelvic infections, bacteremia, endocarditis and meningitis. *Enterococcus faecalis* is responsible for about 80-90% of all enterococcal infections [1]. Severe enterococcal infections require a bactericidal therapy, which is usually a combination of a cell wall active agent such as ampicillin, penicillin or a glycopeptide plus gentamicin or streptomycin. However, the increasing resistance to high levels of aminoglycosides (HLAR) and to other antimicrobials leads to the loss of effective synergisms and thus bactericidal activity [1, 2].

Enterococci are considered to be resistant to cephalosporins. Nonetheless, a synergistic effect between penicillin and cephalosporins has been reported in several in vitro studies. Mainardi observed an in vitro synergism between amoxicillin and cefotaxime in 48 out of 50 *E. faecalis* strains due to the saturation of PBPs 2 and 3 by cefotaxime in addition to the partial saturation of PBPs 4 and 5 with amoxicillin [3]. In another experiment, a synergistic effect between the two antimicrobials was not obtained in *E. faecalis* rabbit endocarditis because of the brief period of time in which amoxicillin and cefotaxime concentrations remained in the range required to obtain synergism [4]. Gavaldà demonstrated a synergistic effect between ampicillin and ceftriaxone in vitro as well as in an experimental model of *E. faecalis* endocarditis [5-7]. The same author reported at three months an overall cure rate of 67.4% among 43 patients with *E. faecalis* endocarditis, 21 HLAR and 22 non-HLAR [6]. Others reported controversial results [8, 10].

Enhanced bactericidal activity has been shown for ampicillin combined with imipenem in *E. faecium* experimental endocarditis [11]. Ertapenem is another carbapenem antibiotic with a broad spectrum of antibacterial activity against anaerobic and aerobic Gram negative bacteria, excluding the non-fermenters, and anaerobic and aerobic Gram positives, excluding methicillin resistant *Staphylococcus aureus* and enterococci [12-14]. In contrast to imipenem, it is less active against *Pseudomonas *spp., *Acinetobacter* spp. and Gram-positive pathogens due to the poor affinity of the PBPs of these micoorganisms [12-14].

The purpose of this work was to further analyse the in vitro interaction between amipicillin and ceftriaxone and to evaluate that of ampicillin-ertapenem combination against five high-level aminoglycoside resistant *E. faecalis* strains isolated from patients with bacteremia.

## METHODS

### Bacterial strains.

Five strains of *E. faecalis* isolated from bacteremic patients were studied. Bacterial identification was verified by standardized procedures (API 20 Strep, Bio-Merieux, France). Strains were stored at -70°C until use. Before each experiment aliquots were thawed and sub-cultured in 5% sheep Columbia agar plates.

### Antibiotic susceptibility studies.

Beta-lactamase production was assessed with the nitrocephin disk test (Becton Dickinson, Sparks, Maryland, USA). Antibiotic susceptibility was determined by agar diffusion [15]. Etest (AB-BIODISK, Solna, Sweden), and with the Phoenix microdilution (Becton Dickinson) methods. For ampicillin, ceftriaxone and ertapenem MICs and MBCs were determined also using the standard macrodilution method in Müller-Hinton broth (MHB) with a bacterial inoculum of 2-5x10^5^ CFU/ml [16-17]. Stock solutions of ampicillin, ceftriaxone or ertapenem were prepared and stored at -20°C until the day of use. To determine bactericidal activity of antibiotics 0.1 ml of the bacterial suspensions from each dilution tube were sub-cultured in Müller-Hinton agar (MHA) plates [18].  Plates were incubated at 37°C for 24 and 48 hours and colonies were counted at the same times. An effect was considered bactericidal when killing of ≥10^3^ CFU/ml from the initial inoculum was recorded [18]. *E. faecalis* ATCC 29212 was used as control strain.

### Synergy studies.

A qualitative estimation (bacteriostatic activity) of the synergy between ampicillin and ceftriaxone or ampicillin and ertapenem was first evaluated utilizing the double disk method on MHA plates [19]. The disk antibiotic concentrations were as follows: ampicillin, 10 μg (BioMerieux); ceftriaxone, 30 μg (BioMerieux); ertapenem, 10 μg (Becton Dickinson).

Time killing studies were performed in MHB supplemented with several concentrations of ampicillin, ceftriaxone, and ertapenem [5, 19]. The final antibiotic concentrations were the following: ampicillin 0.5, 1, 2 and 4 mg/l (between MIC and sub-inhibitory concentrations); ceftriaxone 10, 20, 40 and 80 mg/l, between peak and trough values during patient treatment; ertapenem 2 mg/l, trough value. The bactericidal activity was evaluated for ampicillin alone or in combination with ceftriaxone or ertapenem. Test tubes were inoculated with a suspension of enterococcal strains at a final concentration of 2-5x10^5^ CFU/ml or 2-5x10^7^ CFU/ml and incubated at 37°C. A tube without antibiotics was used as positive growth control. The number of CFU/ml was obtained at time 0, after 4 and 24 hours of incubation plating on MHA a number of different dilutions from each tube. Data are the mean of CFU recorded for each plate. To reduce the carry over effect the inoculum was spread over the entire plate surface after the broth had dried [19].

To define antimicrobial interactions the criteria previously applied [5, 20] have been utilized. Antimicrobial interactions were defined as additive when a ≥ 2 log_10_ CFU/ml decrease between the combination and its most active agent alone was registered after 24 hr. If the reduction reached ≥ 3 log_10_ the combination was considered to have a synergistic effect [5, 20].

## RESULTS

The five *E. faecalis* strains in the study were isolated from blood. Three were from patients with central line related sepsis, one from a patient with a liver abscess and another from a patient with prosthetic valve endocarditis (Table **1**). All the five strains were beta-lactamase negative. MIC values indicate that they were susceptible to ampicillin, linezolid, glycopeptides and resistant to ceftriaxone and ertapenem (Table **1**). All the isolates were highly resistant to aminoglycosides. Isolate 2927 had gentamicin MIC >500 mg/l, and streptomycin MIC 64 mg/l, while strain 2980 had gentamicin and streptomycin MIC respectively 8 mg/l and >1000 mg/l. The other three strains were both gentamicin and streptomycin resistant (Table **1**).

After 24 hours of incubation MBCs of ampicillin varied from 4 to above 32 mg/l (Table **2**), producing a bactericidal effect (99.9% killing of the initial bacterial inoculum) in four out of five strains. Only one strain (2980) appeared to be tolerant to ampicillin. Intermediate concentrations of ampicillin (2 and 4 mg/l) produced more efficacious bacterial killing for strains 2927 and 2980 (Table **2**). MICs of ceftriaxone and ertapenem were consistent with the resistance break points, therefore sub-cultures for MBC evaluations were not obtained.

A bacteriostatic synergistic effect between ampicillin and ceftriaxone, recorded with two + in Table **2**, was observed with the disk diffusion assay for three of the five enterococcal strains evaluated (Table **3**). In broth, ampicillin MICs did not change or decreased of only one tube dilution when either ceftriaxone or ertapenem at concentrations of 10 mg/l and 2 mg/l, respectively, were added (Table **3**).

The time killing curves of ampicillin alone or in combination with 10 mg/l ceftriaxone showed after four hours of contact that 4 mg/l ampicillin produced a bacteriostatic effect with colony counts ranging from minus 0.3 to minus 2.9 log_10_ in all the strains (Table **4**). At the same intervals, a bacteriostatic effect was also observed with 2 mg/l ampicillin for strains 2929 and 2980 and 1 mg/l against strain 2980. Ceftriaxone alone (10 mg/l) did not produce bacteriostatic effect. The addition of ceftriaxone (10 mg/l) to ampicillin did not modify the antibacterial activity of ampicillin for strains 2557 and 2648 either after 4 or 24 hr. On the contrary, a positive bactericidal interaction resulted for strains 2927, 2929 and 2980. Indeed, the combination 4 mg/l ampicillin-ceftriaxone produced a bactericidal effect for strains 2927 and 2980, 2 mg/l ampicillin-ceftriaxone for strains 2929 and 2980, 1 mg/l ampicillin-ceftriaxone for strain 2929. Overall, after 24 hr a synergism was recorded for the combinations 4 mg/l, 1 mg/l, 2 mg/l ampicillin-ceftriaxone against strains 2927, 2929 and 2980 respectively, while an additive effect was seen with the combinations 0.5 mg/l ampicillin-ceftriaxone against isolates 2927, 2929, and 2 mg/l ampicillin-ceftriaxone against strain 2927. Using an higher bacterial inoculum (10^7 ^CFU/ml) the synergism ampicillin-ceftriaxone was still detected only with 2 mg/l ampicillin for the isolate 2980 (data not shown). The synergism between ampicillin-ceftriaxone was not affected when 2 mg/l of ampicillin was combined with increasing ceftriaxone concentrations (Table **4**).

In all the experiments the bactericidal activity of ampicillin was never decreased by ceftriaxone.

When ampicillin was combined with 2 mg/l of ertapenem non consistent positive or negative interactions were registered against the strains tested (Table **4**).

## DISCUSSION

High levels of gentamicin and streptomycin resistances preclude bactericidal synergism with penicillin, ampicillin or glycopeptides for serious enterococcal infections. In these clinical situations other antimicrobial associations [2-11] have been evaluated but, given that their clinical data have not been fully conclusive, further investigations are needed. At the same time, other new additional associations need to be explored. Enterococci are considered intrinsically resistant to cephalosporins [1, 2, 15]. Ertapenem is also reported to be inactive against enterococci [12-14]. Several authors have observed enhanced in vitro bactericidal activity of ampicillin combined with cephalosporins [3-8]. Other researchers have observed enhanced activity of the combination ampicillin-imipenem against *E. faecium* in experimental endocarditis [11].

In this study, the antimicrobial activity of ampicillin alone and in combination with ceftriaxone or ertapenem in five strains of high-level gentamicin and streptomycin resistant *E. faecalis* isolated from bacteremic patients was evaluated by both double disk diffusion and time killing methods.

With the broth macromethod MBC test, ampicillin alone produced a bactericidal effect (99.9% killing of the initial bacterial inoculum) in four out of five strains at concentrations equal or above those used for time killing experiments. Only one strain appeared to be tolerant to ampicillin. It is widely known that most enterococci are less likely killed at elevated concentrations of beta-lactam antibiotics [5, 9, 19]. Thus, the bactericidal effect of ampicillin in these experiments may have been due to the tested ampicillin concentrations which were very close to the MICs. Consequently, we may have been unable to evaluate the ampicillin tolerance of these strains [19]. Nevertheless, the purpose of the study was not to evaluate the ampicillin tolerance but rather to evaluate if any in vitro synergistic activity existed between ampicillin and ceftriaxone or ampicillin and ertapenem for these isolates.

Coming to this point, in broth MIC of ampicillin did not changed when a 10 mg/l concentration of ceftriaxone was added. On the contrary an increased antimicrobial activity of ampicillin and ceftriaxone was evidenced with the double disk test and the time killing curves in three out of five strains. With this last technique, non bactericidal concentrations of ampicillin produced an effective bacterial killing when ceftriaxone was added and some of the ampicillin-ceftriaxone combinations showed a real synergistic interaction. Other researchers have shown that saturation of different bacterial PBPs with ampicillin and ceftriaxone is responsible for the enhanced bacterial killing of this antimicrobial combinations [5] and that the effect was persistent for all the isolates tested [5]. Further experiments with an increased number of HLAR *E. faecalis* isolates will help to confirm strain variability to the ampicillin-ceftriaxone combination. More over, better standardized definitions of antimicrobial interactions will help to reduce variability among different studies [21].

All together, the results obtained on ampicillin-ceftriaxone interactions with this small investigation broadened the evidence of bacteriostatic and bactericidal synergism in vitro between ampicillin and ceftriaxone. This effect was evident only for three of the five strains evaluated and was not influenced by the ceftriaxone concentrations. Until further in vitro and clinical data are collected, this combination could be used only in selected clinical situations such as infections caused by highly antimicrobial resistant *E. faecalis* or the occurrence of treatment adverse events [10].

The data on ampicillin-ertapenem combination did not show in vitro synergism either with disk diffusion and time killing curves when an ertapenem concentration of 2 mg/l (concentration observed at trough during patient treatment) was added to ampicillin for all the five strains in the study. Given this, the bactericidal interactions with ertapenem concentrations higher than 2 mg/l was not investigated. Differences in ertapenem affinity for enterococcal PBPs may be a reason for the absence of antibacterial activity of this combination [22-25].

In conclusion, the results of this in vitro study broadened the evidence of bacteriostatic and bactericidal synergism in vitro between ampicillin and ceftriaxone. The effect was evident only for three of the five strains evaluated and was not influenced by the ceftriaxone concentrations. Double disk diffusion was predictive of time killing curve results.

The study has been presented in part at the Infectious Diseases Accademy (IDA), Riunioni delle Scuole di Specializzazione in Malattie Infettive e Tropicali, Udine, 5-6 Luglio 2007.

## Figures and Tables

**Table 1.** Antimicrobial Susceptibility of Five *Enterococcus faecalis* Clinical Isolates

		MIC (mg/l)							
Strain	Diagnosis	A	L	V	T	C	E	G	S
2557	Liver abscess	4	2	2	≤1	>256	32	>500	>1000
2648	CVC^a^ related sepsis	4	2	4	≤1	>256	64	>500	>1000
2927	CVC related sepsis	1	2	2	≤1	>256	32	>500	64
2929	Endocarditis	1	2	2	≤1	>256	16	>500	>1000
2980	CVC related sepsis	2	2	4	≤1	>256	16	8	>1000

A, ampicillin; C, ceftriaxone; E, ertapenem; G, gentamicin; L, linezolid; S, streptomycin; T, teicoplanin; V, vancomycin^a^, central venous catheter

**Table 2.** Activity of Different Ampicillin Concentrations Against Five Isolates *Enterococcus faecalis* Highly Resistant to Aminoglycosides

Ampicillin Concentrations (mg/l)									
Strain	Ampicillin MIC (mg/l)	Ampicillin MBC (mg/l)	0.5	1	2	4	8	16	32
2557	4	16	>2	>2	>2	-0.3	-2.3	**-4.0**	**-4.0**
2648	4	8	>2	>2	>2	-1.5	**-3.0**	**-3.2**	**-3.8**
2927	2	32	>2	>2	**-4.0**	**-3.9**	**-2.9**	**-2.4**	**-3.4**
2929	1	4	>2	-1.9	-1.9	**-3.8**	**-3.8**	**-3.8**	**-3.8**
2980	2	>32	>2	>2	**-3.0**	**-3.2**	-2.6	-2.6	-2.6

Values are change in log _10_CFU/ml

**Table 3.** Bacteriostatic Interactions Between Ampicillin Plus Ceftriaxone or Ertapenem Against Five Isolates of *Enterococcus faecalis* Highly Resistant to Aminoglycosides Using the Double Disk Diffusion and the in Broth MIC Methods

				Disk Diffusion Synergism		MIC (mg/l)	
Strain	A	C	E	A+C	A+E	A+C^a^	A+E ^b^
2557	4	>256	32	-	-	2	2
2648	4	>256	64	-	-	2	2
2927	1	>256	32	++	-	2	2
2929	1	>256	16	++	-	0.5	0.5
2980	2	>256	16	++	-	2	2

A, ampicillin; C, ceftriaxone; E, ertapenem.

^a^, MIC of ampicillin in Muller Hinton Broth with a fixed ceftriaxone concentration (10 mg/l).

^b^, MIC of ampicillin in Muller Hinton Broth with a fixed ertapenem concentration (2 mg/l).

**Table 4.** Results of Time;Killing Experiments with Five Isolates Enterococcus faecalis Highly Resistant to Aminoglycosides: Change in log_10_ CFU/ml from Initial Inoculum (2-5X10^5^ CFU/ml)

Antimicrobials (mg/l)	*Enterococcus faecalis* Strains									
	2557		2648		2927		2929		2980	
	4 h	24 h	4h	24h	4h	24h	4h	24h	4h	24h
None	+1.2	+3.4	+1.2	+3	+2.5	+2.8	+0.6	+3	+2.5	+3.2
										
A(0.5)	+0.8	+2.5	+1.4	+2.3	+2.4	+2.6	+0.3	+1.8	+0.3	+3
A(1)	+0.8	+2.2	+1.4	+2.3	+2.1	+2.6	0	+1.1	-0.4	+1.9
A(2)	+0.6	+1.5	+0.5	+1.3	+0.7	+0.4	-0.9	-2.5	-0.7	+1.5
A(4)	-0.3	-0.2	-0.9	-1	-2	-0.3	-1.9	-3.5	-1.8	-2.4
										
C(10)	+1.2	+3.7	+0.8	+2.9	+1.1	+3	+0.5	+2.2	-0.2	+2.6
										
A(0.5)+C(10)	+1	+2.3	+1.5	+2.1	+0.6	+0.4	-0.9	-0.7	-1.7	+2.6
A(1)+C(10)	+0.8	+1.9	+1.5	+1.7	-0.9	+1.4	-1.2	-3.4	-2.5	+2.4
A(2)+C(10)	+0.7	0	+0.1	+1.1	-2.9	-1.6	-1.6	-3	-2.9	-4
A(4)+C(10)	-0.5	-0.6	-0.6	-0,9	-2.7	-3.7	-2.2	-3.8	-2.9	-3.4
										
A(2)+C(20)	-0.5	+1.4	+0.6	+2	-2	-2.1	-1.2	-3.6	-2.3	-3
A(2)+C(40)	-0.5	+1.2	+0.6	+1.5	-2	-2.3	-2	-3.7	-2.2	-3.1
A(2)+C(80)	-0.8	+2	+0.6	+2.2	-2	-3	-1	-4	-3.3	-3.5
										
E(2)	+0.9	+2.6	+1.8	+2.2	+2.1	+2.7	+0.2	+2.2	+1.8	+2.9
										
A(0.5)+E(2)	+0.6	+1.8	+1.7	+1.9	+1.9	+2.4	-0.5	+1.8	+2.1	+2.6
A(1)+E(2)	+0.3	+1.2	+1.4	+1.6	+1.4	+1.4	-0.9	-0.9	+1.8	+3
A(2)+E(2)	-0.2	+0.4	+0.9	+0.5	-0.6	+0.9	-1.2	-3	-1.8	+2.4
A(4)+E(2)	-0.8	+0.1	-0.7	-0.7	-2	-0.4	-1.2	-3	-2.2	-2.9

A, ampicillin; C, ceftriaxone; E, ertapenem. Numbers in parenthesis are mg/l. Values are change in log_10_ CFU/ml from initial inoculum. In bold concentrations showing bactericidal activity (decrease ≥3 log_10_).

## References

[1] Simonsen GS, Smabrekke L, Monnet DL (2003). Prevalence of resistance to ampicillin, gentamicin and vancomycin in *Enterococcus faecalis* and *Enterococcus faecium* isolates from clinical specimens and use of antimicrobials in five nordic hopsitals. J Antimicrob Chemother.

[2] Babola C, Patel KB, Nightingale CH, Nicolau DP (2004). Synergistic activity of vancomycin and teicoplanin alone and in combination with streptomycin against Enterococcus faecalis strains with various vancomycin susceptibility. Int J Antimicrob Agents.

[3] Mainardi J-L, Gutmann L, Acar JF, Goldstein FW (1995). Synergistic effect of amoxicillin and cefotaxime against *Enterococcus faecalis*. Antimicrob Agents Chemother.

[4] Join-Lambert O, Mainardi J-L, Cuvelier C (1998). Critical importance of in vivo amoxicillin and cefotaxime concentrations for synergy in treatment of experimental *Enterococcus faecalis* endocarditis. Antimicrob Agents Chemother.

[5] Gavalda’ J, Torres C, Tenorio C (1999). Efficacy of ampicillin plus ceftriaxone in treatment of experimental endocarditis due to *Enterococcus faecalis* strains highly resistant to aminoglycosides. Antimicrob Agents Chemother.

[6] Gavalda’ J, Lopez Onrubia P, Martin Gomez MT (2003). Efficacy of ampicillin combined with ceftriaxone and gentamicin in the treatment of experimental endocarditis due to *Enterococcus faecalis* with no high-level resistance to aminoglycosides. J Antimicrob Chemother.

[7] Galvadà J, Len O, Mirò JM (2007). Brief communication treatment of Enterococcus faecalis endocarditis with ampicillin plus ceftriaxone. Ann Intern Med.

[8] Tascini C, Doria R, Leonildi A, Martinelli C, Menichetti F (2004). Efficacy of the combination ampicillin plus ceftriaxone in the treatment of a case of enterococcal endocarditis due to *Enterococcus faecalis* highly resistant to gentamicin efficacy of the *“ex vivo”* synergism method. J Chemother.

[9] Venditti M, Brandimarte C, Capone A (1997). Endocarditis caused by *Enterococcus faecalis* high-level resistant to aminoglycosides failure of ampicillin and ceftriaxone combined therapy. Clin Microbiol Infect.

[10] Baddour LM, Wilson WR, Bayer AS (2005). AHA Scientific statement, infective endocarditis diagnosis antimicrobial therapy and management of complications. Circulation.

[11] Brandt CM, Rouse MS, Laue NW, Stratton NW, Wilson WR, Steckelberg JM (1996). Effective treatment of multidrug-resistant enterococcal experimental endocarditis with combination of cell wall active agents. J Infect Dis.

[12] Shah PM, Isaac RD (2003). Ertapenem, the first of a new group of carbapenems. J Antimicrob Chemother.

[13] Fuchs PC, Barry AL, Brown SD (2001). *In vitro* activities of ertapenem (MK-0826) against clinical bacterial isolates from 11 North American Medical Centers. Antimicrob Agent Chemother.

[14] Livermore DM, Carter MW, Bagel S (2001). *In vitro* activities of ertapenem (MK-0826) against recent clinical bacteria collected in Europe and Australia. Antimicrob Agents Chemother.

[15] Clinical and Laboratory Standard Institute (2006). Performance Standards for Antimicrobial Disk Susceptibility Tests. Approved Standard.

[16] Clinical and Laboratory Standard Institute (2006). Dilution Antimicrobial Susceptibility Tests for Bacteria That Grow Aerobically. Approved Standard.

[17] Clinical and Laboratory Standard Institute (2006). Performance Standards for Antimicrobial Susceptibility Testing.

[18] Clinical and Laboratory Standard Institute (1999). Methodology for the Serum Bactericidal Test. Approved Guideline.

[19] Stratton CW, Cooksey RC, Balows A, Hausler WJ, Herrmann KL, Isenberg HD, Shadomy HJ (1991). Susceptibility test: special tests. Manual of Clinical Microbiology.

[20] Cedric J, Navas D, Batard E (2005). *In vitro* and *in vivo* synergistic activities of linezolid combined with sub inhibitory concentrations of imipenem against methicillin-resistant *Staphylococcus aureus*. Antimicrob Agents Chemother.

[21] Desbiolles N, Piroth L, Lequeu C, Neuwirth C, Portier H, Chavanet P (2001). Fractional maximal effect method for in vitro synergy between amoxicillin and ceftriaxone and between vancomycin and ceftriaxone against *Enterococcus faecalis* and penicillin-resistant *Streptococcus pneumoniae*. Antimicrob Agents Chemother.

[22] Ono S, Muratani T, Matsumoto T (2005). Mechanisms of resistance to imipenem and ampicillin in *Enterococcus faecalis*. Antimicrob Agents Chemother.

[23] Fontana R, Canepari P, Lleo MM, Satta G (1990). Mechanisms of resistance of enterococci to beta-lactam antibiotics. Eur J Clin Microbiol Infect Dis.

[24] Fontana R, Ligozzi M, Pittalunga F, Satta G (1996). Intrinsic penicillin resistance in enterococci. Microb Drug Resist.

[25] El Amin N, Lund B, Tjernlund A, Lundberg C, Jalakas C, Wretlind B (2001). Mechanisms of resistance to imipenem in imipenem-resistant ampicillin-sensitive *Enterococcus faecium*. APMIS.

